# Vismodegib Suppresses TRAIL-mediated Liver Injury in a Mouse Model of Nonalcoholic Steatohepatitis

**DOI:** 10.1371/journal.pone.0070599

**Published:** 2013-07-22

**Authors:** Petra Hirsova, Samar H. Ibrahim, Steven F. Bronk, Hideo Yagita, Gregory J. Gores

**Affiliations:** 1 Division of Gastroenterology and Hepatology, Mayo Clinic, Rochester, Minnesota, United States of America; 2 Department of Pharmacology, Faculty of Medicine in Hradec Kralove, Charles University in Prague, Hradec Kralove, Czech Republic; 3 Department of Immunology, School of Medicine, Juntendo University, Tokyo, Japan; Institute of Hepatology, Foundation for Liver Research, United Kingdom

## Abstract

Hedgehog signaling pathway activation has been implicated in the pathogenesis of NASH. Despite this concept, hedgehog pathway inhibitors have not been explored. Thus, we examined the effect of vismodegib, a hedgehog signaling pathway inhibitor, in a diet-induced model of NASH. C57BL/6 mice were placed on 3-month chow or FFC (high saturated fats, fructose, and cholesterol) diet. One week prior to sacrifice, mice were treated with vismodegib or vehicle. Mice fed the FFC diet developed significant steatosis, which was unchanged by vismodegib therapy. In contrast, vismodegib significantly attenuated FFC-induced liver injury as manifested by reduced serum ALT and hepatic TUNEL-positive cells. In line with the decreased apoptosis, vismodegib prevented FFC-induced strong upregulation of death receptor DR5 and its ligand TRAIL. In addition, FFC-fed mice, but not chow-fed animals, underwent significant liver injury and apoptosis following treatment with a DR5 agonist; however, this injury was prevented by pre-treatment with vismodegib. Consistent with a reduction in liver injury, vismodegib normalized FFC-induced markers of inflammation including mRNA for TNF-α, IL-1β, IL-6, monocyte chemotactic protein-1 and a variety of macrophage markers. Furthermore, vismodegib in FFC-fed mice abrogated indices of hepatic fibrogenesis. In conclusion, inhibition of hedgehog signaling with vismodegib appears to reduce TRAIL-mediated liver injury in a nutrient excess model of NASH, thereby attenuating hepatic inflammation and fibrosis. We speculate that hedgehog signaling inhibition may be salutary in human NASH.

## Introduction

With the increasing prevalence of obesity, nonalcoholic fatty liver disease has become the most common form of chronic liver disease in Western countries [1]. A subset of patients with nonalcoholic fatty liver disease progress to nonalcoholic steatohepatitis (NASH), a more aggressive form of the disease characterized by hepatocyte apoptosis, hepatic infiltration by inflammatory cells and fibrosis, which may culminate in liver cirrhosis and the development of hepatocellular carcinoma. Unfortunately, there is no proven therapy for NASH, and lifestyle modifications remain the standard of care which are often difficult to obtain and sustain (e.g., weight loss).

Hepatocellular apoptosis appears to be a cellular mechanism distinguishing simple fatty liver disease from NASH [2]. Indeed, the extent of hepatocyte apoptosis differs significantly between simple steatosis and NASH, and excessive hepatocyte cell death is a pathologic hallmark of NASH. Apoptosis can be executed by two general pathways: intrinsic (organelle-initiated) and extrinsic (death receptor-mediated) pathways. Although regulation of apoptosis during liver injury is very complex, death receptor-mediated apoptosis plays a major role in NASH [3], [4]. Death receptors important for liver injury include Fas, tumor necrosis factor (TNF) receptor 1 (TNFR1), and death receptors 4 (DR4, TRAIL receptor-1) and 5 (DR5, TRAIL receptor-2). Death receptor-mediated apoptosis is triggered when ligands TNF-α, tumor necrosis factor-related apoptosis-inducing ligand (TRAIL) and Fas ligand bind their cognate death receptors TNFR1, DR4/5 and Fas, respectively, activating downstream death-inducing cell signaling cascades. The ligands for these receptors are expressed by cells of the immune system, TNF-α and TRAIL by cells of the innate immune system such as NK cells and macrophages, and Fas ligand by T-lymphocytes [5], [6]. Modulation of hepatocyte apoptosis by death receptors, especially as mediated by inflammatory cells, is a potential therapeutic strategy for NASH, but has yet to be realized.

Hedgehog signaling pathway plays a key role not only in embryonic development but also in tumorigenesis, repair and regeneration in adult tissues. The canonical hedgehog signaling cascade is initiated by binding hedgehog ligands (e.g., sonic, indian and desert hedgehog) to the plasma membrane receptor patched. Activation of patched disinhibits the plasma membrane receptor smoothened, which allows for nuclear translocation of glioma-associated oncogene (Gli) transcription factors. These can both activate and inhibit expression of their target genes (e.g., patched 1). Besides the canonical pathway, non-canonical signaling cascades have been described. These pathways also require smoothened but do not involve Gli-mediated transcription responses [7]. Aberrant activation of hedgehog signaling has been observed in both murine and human NASH [8], [9], [10], [11]. In animal models of NASH, hedgehog signaling promotes hepatic fibrogenesis [10], [12]. Despite the substantial body of evidence that hedgehog signaling plays an important role in NASH progression, therapeutic inhibition of this pathway has not been examined in animal model of NASH. In particular, the role of hedgehog signaling pathway in modulating hepatocyte apoptosis has yet to be explored.

In the present study, we examined the hypothesis that inhibition of hedgehog signaling in NASH has a salutary effect on the disease by decreasing liver injury. We observed that vismodegib, a clinically used smoothened inhibitor, attenuates liver cell apoptosis via downregulation of DR5 and also reduces macrophage-associated hepatic inflammation in a dietary mouse model of NASH.

## Materials and Methods

### Animals and experimental design

All animal studies were performed in accord with and approved by the Institutional Animal Care and Use Committee at the Mayo Clinic. C57BL/6 mice (8 wk of age) were obtained from the Jackson Laboratory (Bar Harbor, ME) and housed with a 12-h light-dark cycle. Mice were fed either a standard chow or a diet high in saturated fat and cholesterol with exogenous glucose plus fructose added to the drinking water [13]; we have termed this diet the FFC (high saturated fat, high fructose, and high cholesterol) diet. This model of diet-induced obesity results in the development of murine NASH with liver inflammation, ballooned hepatocytes, and fibrosis and mimics human NASH [13]. Mice were fed either the standard chow or FFC diet for three months. In one paradigm, mice (5–7 mice per group) fed either the chow or the FFC diet were treated with vismodegib (25 mg/kg body wt) or vehicle (equal volume of 0.5% carboxymethylcellulose) once daily by gastric gavage for 7 consecutive days prior to sacrifice. For the second paradigm, mice were fed the standard chow or FFC diet for three months (3–4 per group), and treated with either vismodegib or vehicle by gastric gavage once daily for 14 days. On day 7 and 10 of the treatment with vismodegib or vehicle, MD5-1, an agonistic antibody against mouse DR5 [14], was administered i.p. (300 µg/animal) to a group of vismodegib- and vehicle-treated mice on each diet. At the end of the treatment mice were sacrificed under general anesthesia induced by pentobarbital (50 mg/kg body wt, i.p.), blood and liver samples were collected for further analysis.

### Primary cell isolation and cell culture

For hepatocyte and liver macrophage isolation, mice were fed standard chow or FFC diet for three months (3–5 per group) and treated with either vismodegib (25 mg/kg body wt) or vehicle (equal volume of 0.5% carboxymethylcellulose) once daily by gastric gavage for 7 consecutive days prior to primary cell isolation. Hepatocytes and macrophages were isolated by collagenase perfusion and purified by Percoll (Sigma, St. Louis, MO) gradient centrifugation as previously described by us [15]. Hepatocytes were immediately snap frozen for RNA and protein extraction. Macrophages were plated and allowed to attach to a culture dish for 30 min, thereafter attached cells were harvested for RNA isolation. Huh-7, a human hepatocellular carcinoma cell line, was cultured in Dulbecco's modified Eagle's medium containing glucose (4.5 g/L), penicillin (100 units/mL), streptomycin (100 µg/mL) and 10% fetal bovine serum (all Gibco, Carlsbad, CA). Huh-7 cells were pre-treated with vismodegib (0.01 – 1 µM) or vehicle (DMSO) for 16 h and then treated with palmitic acid (600 µM). Palmitic acid was prepared as described previously by us and the concentration used corresponds to the fasting total free fatty acid plasma concentrations observed in humans with NASH [16].

### Biochemical analysis

Serum ALT activity was determined using a standardized and automated procedure of the diagnostic laboratory of the Mayo Clinic. Liver triglyceride concentrations were measured as described previously [17] and normalized to liver protein concentrations.

### Histopathology, immunohistochemistry, Sirius red staining and terminal deoxynucleotidyl transferase dUTP nick-end labeling assays on liver tissue

For histological review of hematoxylin and eosin (H&E)–stained liver sections by light microscopy (Eclipse Meta Morph V 5.0.7, Nikon, West Lafayette, IN), the liver was diced into 5 mm×5 mm sections, fixed in 4% paraformaldehyde for 48 hours, and then embedded in paraffin. Tissue sections (4 µm) were prepared using a microtome (Reichert Scientific Instruments, Buffalo, NY) and placed on glass slides. H&E staining was performed according to standard techniques. For immunohistochemistry, paraformaldehyde-fixed paraffin-embedded liver tissue sections were deparaffinized, hydrated and incubated with antibodies against Mac-2 (1∶250, eBioscience, San Diego, CA) and sonic hedgehog (Shh, 1∶500, Santa Cruz Biotechnology, Santa Cruz, CA). Bound antibodies were detected using Vectastain ABC kit and diaminobenzidine as a substrate (both Vector Laboratories, Burlingame, CA) and the tissue slices were counterstained with methyl green or hematoxylin. To quantify Mac-2 immunohistochemical staining, ten random pictures of 20× fields per section were assessed by morphometry (KS 400 software, Carl Zeiss). Liver fibrosis was quantified using Sirius red staining. Direct red 80 and Fast-green FCF (color index 42053) were obtained from Sigma-Aldrich Diagnostics. Liver sections were stained, and red-stained collagen fibers were quantified by digital image analysis as previously described by us in detail [18]. The fluorescent terminal deoxynucleotidyl transferase dUTP nick-end labeling (TUNEL) assay on liver tissue (In situ cell death detection kit, Roche, Indianapolis, IN) was performed on frozen liver sections. Briefly, liver tissue samples were cryopreserved in Tissue Tek OTC compound (Takeda, Deerfield, IL) immediately after removal. Tissue sections were cut at 5 µm on a cryomicrotome (Leica, Buffalo Grove, IL), air dried and stored at –80°C until use. The TUNEL assay was then performed using the manufacturer's protocol and tissue slices were mounted with ProLong Gold antifade reagent with DAPI (Life Technologies, Grand Island, NY). Apoptotic cells were quantified by counting TUNEL-positive nuclei in 20 random microscopic fields (20×) using a fluorescent microscope (Eclipse 80i, Nikon, West Lafayette, IN).

### Coherent anti-Stokes Raman scattering and second harmonic generation microscopy

Label-free frozen liver tissue sections (5 µm thick) were imaged on a two photon confocal microscope FluoView FV1000 MPE (Olympus America, Center Valley, PA) using the coherent anti-Stokes Raman scattering (CARS) and second harmonic generation (SHG) applications. Mai Tai DeepSea laser (Spectra-Physics) was tuned to 800 nm and an XLPlanN 25×/1.05w MP objective lens was used. For collagen area quantification, the pixel numbers of the SHG image having intensity above the threshold value were quantified using ImageJ software. Four animals per group and at least ten pictures for each animal were examined.

### Western blot

Whole cell lysates of isolated mouse hepatocytes were obtained using lysis buffer (50 mM Tris-HCl, pH 7.4; 1% Nonidet P-40; 0.25% sodium deoxycholate; 150 mM NaCl; 1 mM EDTA; 1 mM PMSF; 1 µg/mL aprotinin, leupeptin, pepstatin) followed by centrifugation at 15,000×g for 15 min at 4°C to remove cellular debris. Equal amounts of protein (50 µg) were loaded onto SDS-PAGE gel, transferred to nitrocellulose membrane and incubated overnight with anti-smoothened antibody (ab72130, Abcam, Cambridge, MA) at a dilution of 1∶5,000. Horseradish peroxidase-conjugated secondary antibody (SantaCruz Biotechnologies) was used at a dilution of a 1∶5,000. GAPDH protein levels were used as a loading control. Bound antibodies were detected using enhanced chemiluminescence reagent (Amersham, Arlington Heights, IL) and Kodak X-OMAT film.

### Quantitative real-time polymerase chain reaction

Total RNA from liver tissue, primary cells and Huh-7 cells was isolated with RNeasy Plus Kit or RNeasy Micro Kit (Qiagen, Valencia, CA) and was reverse transcribed with Moloney murine leukemia virus reverse transcriptase and oligo-dT random primers (both from Invitrogen, Carlsbad, CA). Quantification of gene expression was performed by real-time polymerase chain reaction (PCR) using SYBR green fluorescence on a LightCycler 480 instrument (Roche Applied, Indianapolis, IN). Specific primers are listed in Table 1. Target gene expression was calculated using ΔΔ Ct method. Expression was normalized to 18S rRNA expression levels, which were stable across the four experimental groups. All data represent fold change over expression in vehicle-treated mice fed standard chow diet.

**Table 1:** Primer sequences for quantitative real-time PCR.

Gene	Forward primer sequence (5′-3′)	Reverse primer sequence (5′-3′)
αSMA	GTC CCA GAC ATC AGG GAG TAA	TCG GAT ACT TCA GCG TCA GGA
ACC1	ATG GGC GGA ATG GTC TCT TTC	TGG GGA CCT TGT CTT CAT CAT
ACOX1	TCC AGA CTT CCA ACAT GAG GA	CTG GGC GTA GGT GCC AAT TA
CD14	CTC TGT CCT TAA AGC GGC TTA C	GTT GCG GAG GTT CAA GAT GTT
CD68	TGT CTG ATC TTG CTA GGA CCG	GAG AGT AAC GGC CTT TTT GTG A
Collagen 1a1	GCT CCT CTT AGG GGC CAC T	CCA CGT CTC ACC ATT GGG G
CPT1a	CTC CGC CTG AGC CAT GAA G	CAC CAG TGA TGA TGC CAT TCT
CPT2	CAG CAC AGC ATC GTA CCC A	TCC CAA TGC CGT TCT CAA AAT
DGAT1	TCC GTC CAG GGT GGT AGT G	TGA ACA AAG AAT CTT GCA GAC GA
DGAT2	GCG CTA CTT CCG AGA CTA CTT	GGG CCT TAT GCC AGG AAA CT
DR5/TRAIL-R2	CGG GCA GAT CAC TAC ACC C	TGT TAC TGG AAC AAA GAC AGC C
Fas	TAT CAA GGA GGC CCA TTT TGC	TGT TTC CAC TTC TAA ACC ATG CT
Fas ligand	TCC GTG AGT TCA CCA ACC AAA	GGG GGT TCC CTG TTA AAT GGG
FASN	GGA GGT GGT GAT AGC CGG TAT	TGG GTA ATC CAT AGA GCC CAG
F4/80	ATG GAC AAA CCA ACT TTC AAG GC	GCA GAC TGA GTT AGG ACC ACA A
Gli1	CCT CCT CCT CTC ATT CCA CA	CTC CCA CAA CAA TTC CTG CT
Gli2	CCC CAT CAC CAT TCA TAA GC	CTG CTC CTG TGT CAG TCC AA
IL-1β	GCA ACT GTT CCT GAA CTC AAC T	ATC TTT TGG GGT CCG TCA ACT
IL-6	TAG TCC TTC CTA CCC CAA TTT CC	TTG GTC CTT AGC CAC TCC TTC
MCP-1	TTA AAA ACC TGG ATC GGA ACCA	GCA TTA GCT TCA GAT TTA CGG G
MTTP	CTC TTG GCA GTG CTT TTT CTC T	GAG CTT GTA TAG CCG CTC ATT
Osteopontin	CTC CAT CGT CAT CAT CAT CG	TGC ACC CAG ATC CTA TAG CC
Patched 1	AAA GAA CTG CGG CAA GTT TTT G	CTT CTC CTA TCT TCT GAC GGG T
SCD1	TTC TTG CGA TAC ACT CTG GTG C	CGG GAT TGA ATG TTC TTG TCG T
Smoothened	GAG CGT AGC TTC CGG GAC TA	CTG GGC CGA TTC TTG ATC TCA
TNF-α	CCC TCA CAC TCA GAT CAT CTT CT	GCT ACG ACG TGG GCT ACA G
TNFR1	CCG GGA GAA GAG GGA TAG CTT	TCG GAC AGT CAC TCA CCA AGT
TRAIL	ATG GTG ATT TGC ATA GTG CTC C	GCA AGC AGG GTC TGT TCA AGA
18S	CGC TTC CTT ACC TGG TTG AT	GAG CGA CCA AAG GAA CCA TA

### Statistical analysis

Data are expressed as the means ± SEM. Differences between groups were compared using one-way analysis of variance followed by Bonferroni post-hoc test. An unpaired t-test was used for comparing two groups. DR5 gene expression in Huh-7 cells was analyzed using one-way analysis of variance followed by Dunnett's test. Differences were considered significant at *P*<0.05. All analyses were performed using GraphPad Prism 5.0 software (San Diego, CA).

### Materials

Vismodegib (GDC-0449) was purchased from Active Biochem (Maplewood, NJ). All other chemicals used were purchased from Fisher Scientific (Suwanee, GA) and Sigma-Aldrich (St. Louis, MO).

## Results

### Hedgehog signaling pathway is activated in a nutrient excess model of NASH

**Figure 1:**
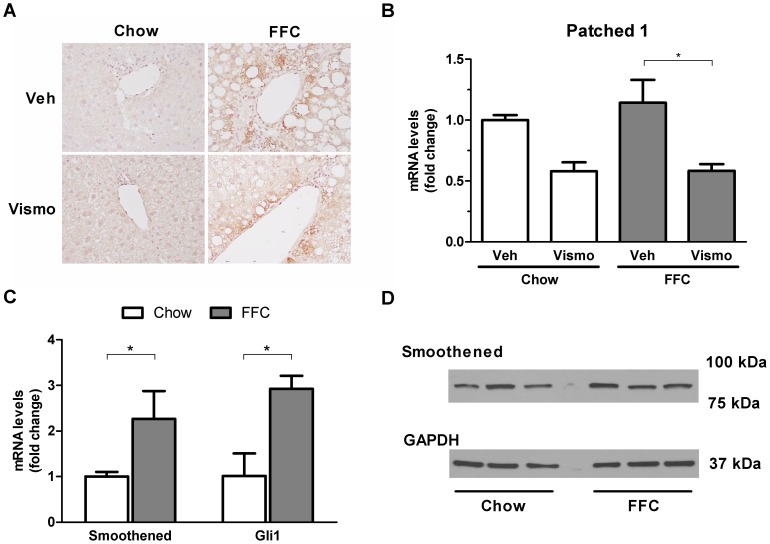
Hedgehog signaling pathway is activated in a nutrient excess model of NASH. C57BL/6 mice were fed chow or the FFC diet for 3 months. Mice were then treated with vismodegib (25 mg/kg body wt) or vehicle for an additional week prior to sacrifice. Liver tissue was procured and processed as described in Materials and Methods. (A) Expression of sonic hedgehog was examined by immunohistochemistry on paraffin-embedded liver tissue and representative microphotographs taken with a 40× objective are shown. (B) Total RNA was extracted from the liver tissue and gene expression of patched 1 was quantified by real-time PCR. (C) Hepatocytes were isolated from mice on chow and the FFC diet. Total RNA was extracted and gene expression of smoothened and Gli1 were assessed by real-time PCR. (D) Hepatocytes were isolated from mice on chow and the FFC diet. Protein expression of smoothened was evaluated by western blotting. Bar columns represent mean ± S.E.M. * *P*<0.05.

Similar to reports in human NASH [8], [11], mice fed the FFC diet displayed expression of sonic hedgehog in the liver (Fig. 1A). While liver sections from mice fed a chow diet were negative for sonic hedgehog, liver tissue from mice on the FFC diet displayed numerous portal/periportal hepatocytes positive for sonic hedgehog by immunohistochemistry. Vismodegib-treated animals on the FFC diet expressed sonic hedgehog to a similar extent as vehicle-treated mice on the FFC diet; however, the mRNA expression of patched 1, an established transcription target of hedgehog signaling [19], was substantially suppressed by the smoothened inhibitor confirming a pharmacologic effect of the inhibition (Fig. 1B). Although smoothened may be expressed in fibroblasts [20], its expression in hepatocytes has not been reported. Therefore, we determined if smoothened is expressed in isolated mouse hepatocytes. Indeed, real-time PCR and immunoblot analysis identified smoothened at both the mRNA and protein levels in these isolated hepatocytes (Fig. 1C, D). The expression of smoothened appears to be coupled to hedgehog signaling as Gli1 expression was 3-fold higher in hepatocytes from FFC-fed as opposed to chow-fed animals (Fig. 1C). Taken together, these data not only demonstrate sonic hedgehog expression in this murine model of NASH analogous to the human disease, but also identify smoothened expression in hepatocytes suggesting these cells may also be capable of manifesting hedgehog signaling pathways.

### Vismodegib treatment suppresses liver injury, but not hepatic steatosis, in mice fed the FFC diet

**Figure 2:**
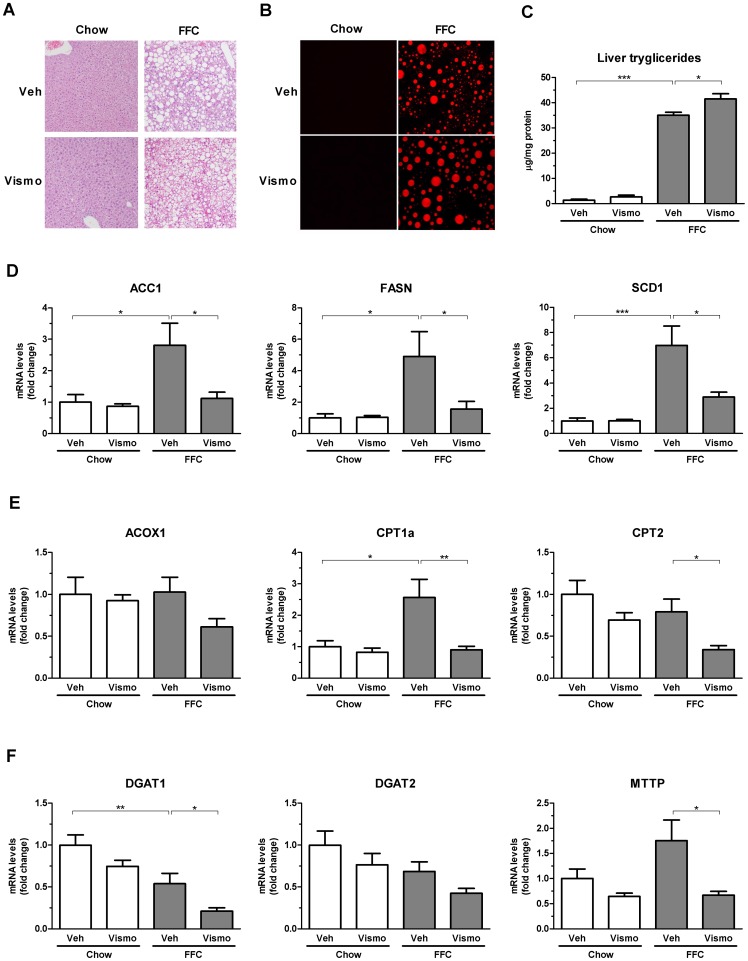
FFC diet induced severe steatosis. Mice were treated as described in Fig. 1. (A) Fixed liver specimens were stained with H&E. (B) Label-free frozen liver tissue sections were imaged by CARS microscopy to visualize steatosis using a 25× objective. (C) Concentration of neutral triglycerides was measured in the liver tissue and normalized to protein concentration. (D-F) Total RNA was extracted from the liver tissue and gene expression of lipogenic enzymes (D), lipolytic enzymes (E) and enzymes involved in triglyceride synthesis and secretion (F) were quantified by real-time PCR. Values are expressed as mean ± S.E.M. *** *P*<0.001, ** *P*<0.05, * *P*<0.01.

After three months on the FFC diet, mice displayed significant hepatic steatosis compared to chow-fed animals as assessed by histology, CARS microscopy, and biochemical quantification of hepatic neutral triglycerides (Fig. 2A, B, and C). Vismodegib therapy did not improve hepatic steatosis as examined by these parameters (Fig. 2A, B and C). In fact, vismodegib treatment in FFC diet-fed animals slightly accentuated triglyceride accumulation in the liver. To provide for the insight into this observation, we examined expression of enzymes involved in lipogenesis, lipolysis and very low density lipoprotein secretion. We evaluated hepatic mRNA levels of key lipogenic enzymes, including acetyl coenzyme A carboxylase-1 (ACC1), fatty acid synthase (FASN), stearoyl coenzyme A desaturase-1 (SCD1), lipolytic enzymes such as peroxisomal acyl-coenzyme A oxidase 1 (ACOX1), carnitine palmitoyltransferase (CPT) 1a and 2. We also assessed mRNA levels of enzymes involved in triglyceride synthesis, such as acyl coenzyme A:diacylglycerol acyltransferase (DGAT) 1 and 2, and expression of microsomal triglyceride transfer protein (MTTP), a key enzyme in very low density lipoprotein assembly and secretion. The FFC diet induced the lipogenic enzymes and CPT1a (Fig. 2D). Somewhat unexpectedly, vismodegib therapy in FFC diet-fed mice reduced expression of lipogenic genes (Fig. 2D). It also reduced key enzymes for mitochondrial fatty acid oxidation (CPT1a and CPT2), but not for peroxisome fatty acid oxidation (ACOX1) (Fig. 2E). As the livers accumulate lipids with vismodegib therapy, presumably the inhibition of MTTP and decreased mitochondrial fatty acid oxidation override the suppression of the lipogenic enzymes.

**Figure 3:**
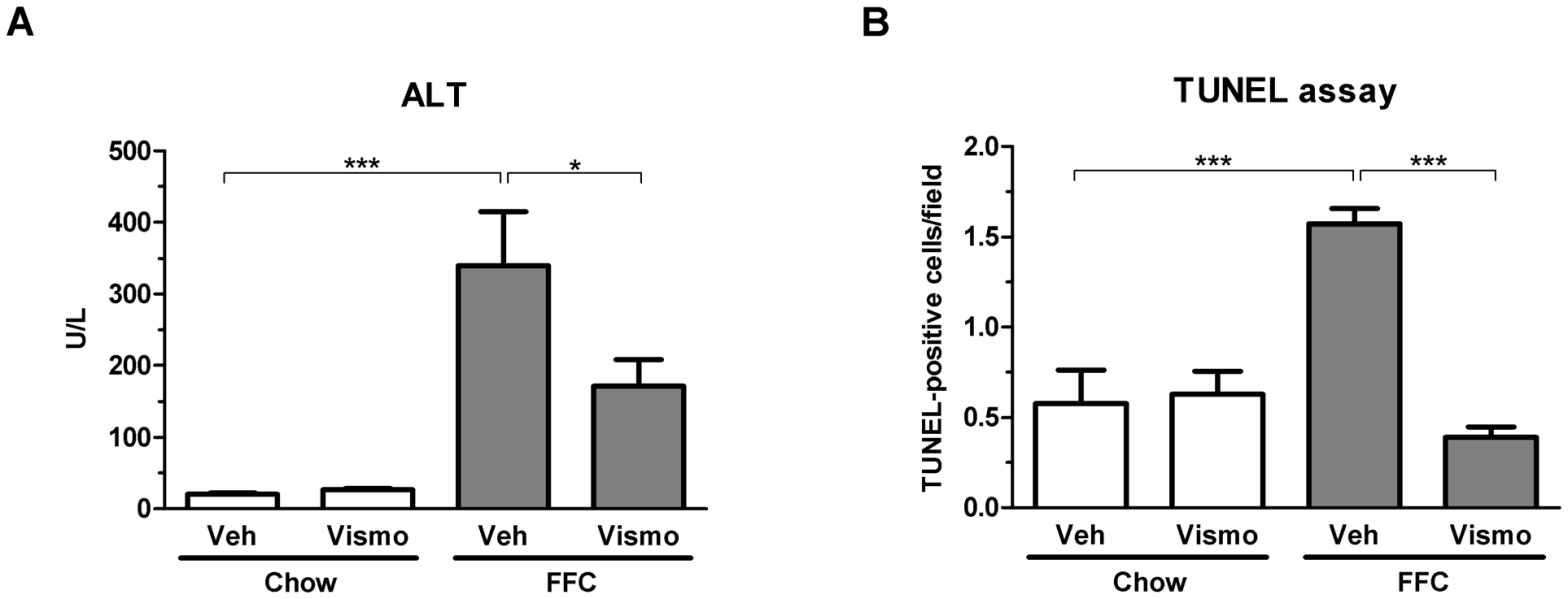
Liver injury is reduced in vismodegib-treated FFC diet-fed mice. (A) Serum ALT values were measured by standard techniques in samples from mice treated as in Fig. 1. (B) Hepatocyte apoptosis was evaluated by the TUNEL assay on frozen liver tissue samples. Apoptotic cells were quantified by counting TUNEL-positive nuclei in 20 random microscopic fields (20×) using a fluorescent microscope. Data represent mean ± S.E.M. *** *P*<0.001, * *P*<0.01.

As previously reported by us, the FFC diet also caused liver injury as manifested by substantially elevated serum ALT values (Fig. 3A). In contrast, vismodegib-treated FFC diet-fed mice displayed reduced serum ALT values compared to vehicle-treated FFC diet-fed animals. As hepatocyte apoptosis is a prominent histopathologic feature of NASH [2], we next examined apoptosis in liver tissue samples using the TUNEL assay. Mice on the FFC diet had an ∼3-fold increase in liver TUNEL-positive cells vs. chow-fed animals (Fig. 3B). Consistent with its effects on serum ALT values, vismodegib also reduced liver TUNEL-positive cells in mice on the FFC diet. These data suggest vismodegib treatment attenuates FFC diet-induced liver injury without altering hepatic steatosis.

### Vismodegib prevents FFC diet-induced upregulation of the murine TRAIL receptor, DR5, and prevents DR5-mediated injury in FFC diet-fed mice

**Figure 4:**
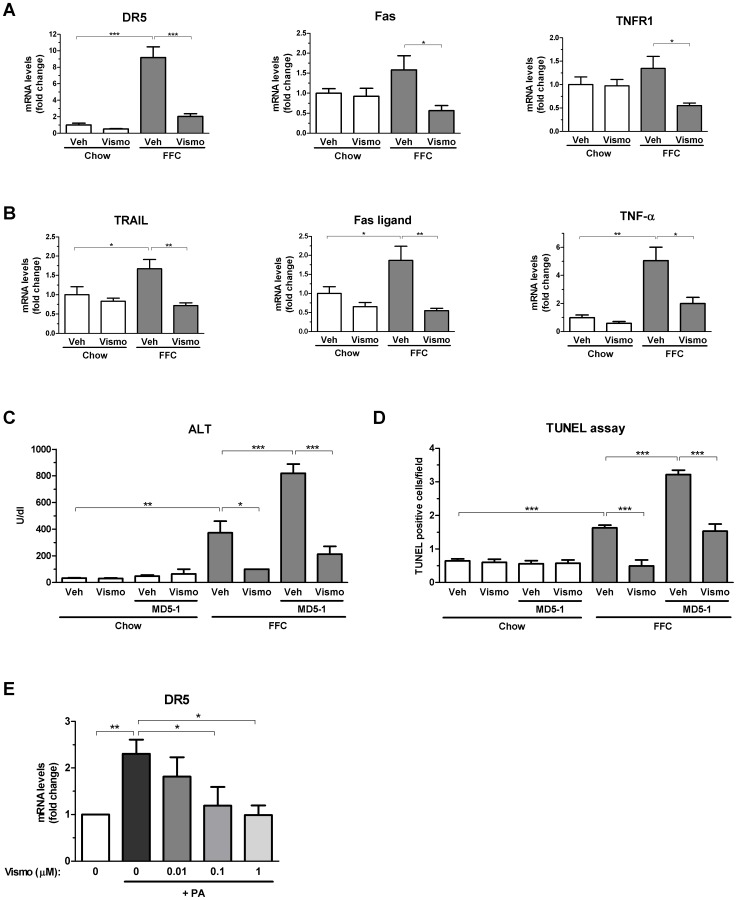
Vismodegib abrogates FFC diet-induced upregulation of death receptor DR5 and prevents DR5-mediated liver injury in FFC diet-fed mice. (A, B) Mice were fed chow or the FFC diet for 3 months. Mice were then treated with vismodegib or vehicle for an additional week prior to sacrifice. Total RNA was extracted from the liver tissue and expression of death receptors (A) and death receptor ligands (B) was quantified by real-time PCR. (C, D) A subset of mice were reared on either chow or FFC diet for 3 months and then treated with vismodegib or vehicle for 2 weeks. Two injections of MD5-1, an agonistic anti-DR5 antibody, were administered to vismodegib- and vehicle-treated groups on each diet during the last week prior to sacrifice. Serum ALT values (C) and liver TUNEL-positive cells (D) were analyzed in all groups. (E) Huh-7 cells were pre-treated with vismodegib (0–1 µM) for 16 h and then treated with 600 µM palmitic acid (PA) for additional 8 h. Total RNA was extracted and DR5 expression was evaluated by real-time PCR. DR5 expression in Huh-7 cells is expressed as mean values of four independent experiments. Data represent mean ± S.E.M. *** *P*<0.001, ** *P*<0.05, * *P*<0.01.

As we detected a decreased rate of apoptosis after vismodegib treatment, we profiled death receptor expression in the liver by real-time PCR. Mice fed the FFC diet had markedly increased hepatic DR5 expression (∼9-fold), while TNFR1 and Fas expression levels were less prominently upregulated (∼1.5-fold) (Fig. 4A). In FFC diet-fed mice, treatment with vismodegib suppressed expression of all three death receptors but the results were most striking for DR5 upregulation. In addition, we assessed mRNA levels of corresponding death receptor ligands. Expression of all TRAIL, Fas ligand and TNF-α were upregulated in FFC diet-fed mice (Fig. 4B). Similar to death receptor mRNA data, vismodegib therapy abrogated these increases. These data suggested the fatty liver was more susceptible to TRAIL-mediated liver injury due to strong DR5 upregulation, a postulate which has been proposed but never directly tested [21]. To more directly test this explanation for our observations, an agonistic anti-DR5 antibody MD5-1 was administered to FFC and chow diet-fed mice treated with vehicle or vismodegib. While administration of MD5-1 to mice on chow diet was not hepatotoxic, the same treatment significantly increased serum ALT values in FFC diet-fed mice (Fig. 4C), suggesting this diet sensitizes mice to DR5-mediated liver injury. Most importantly, vismodegib exerted a strong protective effect by decreasing ALT values in MD5-1-administered FFC diet-fed mice (Fig. 4C). MD5-1 administration also induced hepatocyte apoptosis in FFC diet-fed mice, but not in chow-fed mice, which was reduced by vismodegib pre-treatment (Fig. 4D). Collectively, these data suggest that vismodegib reduces TRAIL:DR-5-mediated liver injury in FFC diet-fed animals.

To test whether vismodegib may directly modulate DR5 expression in hepatocytes, we treated human hepatoma cells (Huh-7) with vismodegib and palmitic acid (600 µM), a known inducer of DR5 [16], and assessed gene expression of the death receptor by real-time PCR. While palmitic acid induced DR5 expression in Huh-7 cells by more than 2-fold, pre-treatment with vismodegib completely abolished this upregulation in a concentration-dependent manner (Fig. 4E). These data suggest that vismodegib may reduce DR5 expression in liver cells. Although Huh-7 cells have been used extensively as a model for lipoapoptosis, we note that there are limitations to using a transformed cell line as a model of normal hepatocyte pathobiology.

### Vismodegib therapy reduces hepatic accumulation of macrophages in mice fed the FFC diet

**Figure 5:**
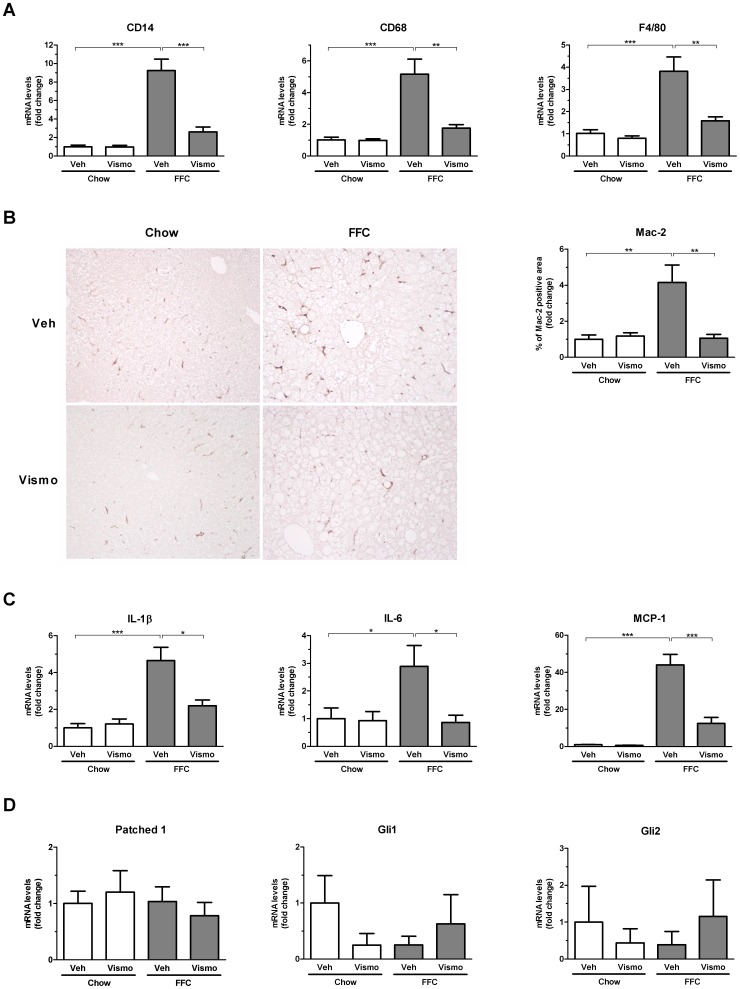
Macrophage accumulation and activation is reduced in vismodegib-treated mice on the FFC diet. (A) Total RNA was extracted from liver tissue obtained from mice treated as described in Fig. 1 and expression profile of several macrophage markers was evaluated by real-time PCR. (B) Another marker of macrophages, Mac-2, was examined by immunohistochemistry on paraffin-embedded liver tissue and representative microphotographs taken with a 20× objective are shown. Macrophage accumulation was assessed by morphometric analysis of Mac-2 positive area in ten random fields per liver tissue section as illustrated in the right panel. (C) Gene expression of cytokines related to macrophage activation, IL-1β, IL-6 and MCP-1, was analyzed by real-time PCR in liver tissue obtained from each experimental group. (D) Liver macrophages were isolated from mice on chow and the FFC diet treated with vehicle or vismodegib. Total RNA was extracted and gene expression of hedgehog signaling target genes were assessed by real-time PCR. Bar columns represent mean ± S.E.M. *** *P*<0.001, ** *P*<0.05, * *P*<0.01.

Several studies have suggested that hepatic lipotoxicity is mediated, in part, by influx and activation of cells of the monocyte lineage within the liver [22], [23]. Moreover, in gastric tissue sonic hedgehog acts as a macrophage chemoattractant [24]. Therefore, we ascertained if cells of this lineage accumulated in the mouse liver on the FFC diet. Quite strikingly, we observed an increase in hepatic mRNA for CD14, CD68, and F4/80 in FFC diet-fed mice, all macrophage markers (Fig. 5A). The substantial accumulation of hepatic macrophages in the FFC diet as compared to chow-fed animals was confirmed by Mac-2 binding protein immunohistochemistry, a marker for phagocytically active macrophages (Fig. 5B). This increase in hepatic macrophage markers was markedly suppressed in FFC diet-fed mice treated with vismodegib (Fig. 5A, B). Evidence for macrophage activation was examined by determining hepatic mRNA expression for TNF-α (Fig. 4B), interleukin (IL)-1β, IL-6 and monocyte chemotactic protein-1 (MCP-1) (Fig. 5C). Indeed, hepatic mRNA for these cytokines and chemokines known to be secreted by activated macrophages were elevated in FFC diet-fed mice, and reduced by treatment with vismodegib. Moreover, apoptotic hepatocytes have recently been demonstrated to release MCP-1, a chemotactic factor for monocytes and macrophages [25], and hence the reduction in cell death (Fig. 3B) likely contributes to the reduction in macrophage accumulation and activation. In addition, to assess the effect of the smoothened inhibitor on hedgehog signaling processes in liver macrophages in our model, we measured mRNA levels of hedgehog target genes in primary macrophages isolated from mice on chow and the FFC diet treated with vehicle or vismodegib. Gene expression of patched 1, Gli1 and Gli2 was not altered by either diet or treatment with the drug (Fig. 5D). However, these data do not preclude an effect of vismodegib on macrophage responses by non-canonical hedgehog signaling pathways. Collectively, the present observations suggest vismodegib suppresses diet-induced liver injury, in part, by reducing infiltration and/or activation of macrophages within the liver.

### Vismodegib therapy attenuates hepatic fibrosis induced by the FFC diet

**Figure 6:**
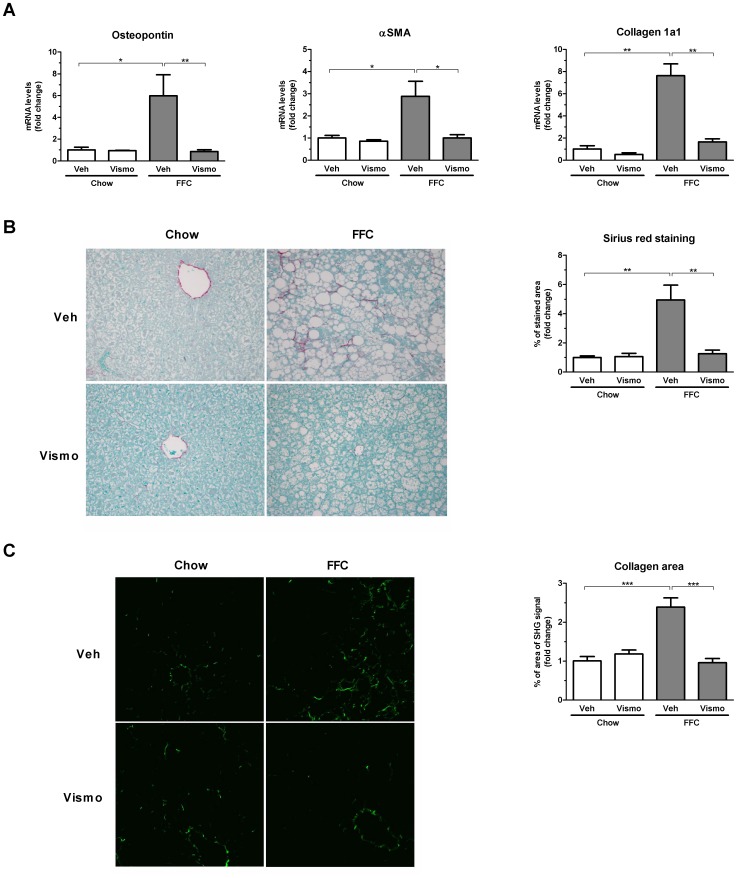
Vismodegib attenuates FFC diet-induced liver fibrosis. (A) Total RNA was extracted from liver tissue obtained from mice treated as described in Fig. 1 and expression profile of profibrogenic markers was evaluated by real-time PCR. (B) Fixed liver tissue sections were stained with Sirius red to detect collagen deposition. Digital pictures of Sirius red staining (taken with a 20× objective) were then assessed by morphometry as indicated in the right panel. (C) Label-free frozen liver tissue sections were imaged by SHG microscopy to visualize collagen deposition using a 25× objective. Collagen area was then quantified as an area of SHG signal having intensity above the threshold value using automated software. Bar columns represent mean ± S.E.M. *** *P*<0.001, ** *P*<0.05, * *P*<0.01.

Hepatic fibrosis is the nefarious consequence of chronic liver inflammation. Therefore, we next examined the effect of vismodegib on NASH-related hepatic fibrogenesis at the levels of gene expression and collagen tissue deposition. Osteopontin, a profibrogenic extracellular matrix protein and cytokine, α smooth muscle actin (αSMA), a marker of activated hepatic stellate cells, and collagen 1a1 mRNA levels were all upregulated by the FFC diet (Fig. 6A). Vismodegib treatment in FFC diet-fed mice abrogated the upregulation of these profibrogenic genes. To assess deposition of excessive collagen matrix, liver sections were stained with Sirius red (Fig. 6B). Liver sections from mice fed the FFC diet had a significantly increased area of collagen deposition compared to mice on chow diet, which was remarkably reduced by vismodegib treatment (Fig. 6B). These data were confirmed by second harmonic generation microscopy for collagen fibrils performed on frozen liver sections (Fig. 6C). These data are consistent with prior observations that hedgehog pathway signaling inhibition by vismodegib or cyclopamine is antifibrogenic in the liver [26], [27].

## Discussion

The principal findings of this study provide mechanistic insights regarding the efficacy of a clinically relevant, pharmacologic smoothened inhibitor vismodegib in a murine model of NASH. Our results indicate the vismodegib treatment in a dietary nutrient excess model of murine NASH has several salutary effects including: (i) a decrease in hepatocyte injury despite lipid loading of the hepatocytes; (ii) inhibition of DR5 upregulation and DR5-mediated liver injury; (iii) a reduction in hepatic markers of macrophage accumulation and activation; and (iv) decreased fibrosis. These observations are more thoroughly discussed below.

In this study we used a nutrient excess model of murine NASH [13]. The model includes a diet high in saturated fats, cholesterol and the addition of fructose in the drinking water and was developed to replicate a western fast food diet. This animal model has been previously published and mimics several features of human NASH including neutral lipid accumulation by hepatocytes, the presence of ballooned hepatocytes, an increase in hepatic inflammatory cells, and liver fibrosis. Although the prior publication reported on mice fed the diet for six months, shorter time periods were not examined. In the current study, mice were fed the diet for three months and at this time point all features of human NASH were present, albeit perhaps not as florid as at the sixth month time period. Interestingly, only one week of vismodegib therapy attenuated all the injurious features of NASH in this model. Whether vismodegib therapy would be equally effective in advanced stages of NASH or following more extended periods of feeding animals a FFC diet remains to be examined. Nonetheless, the results imply a dominant role for hedgehog pathway signaling in the pathogenesis of early NASH, an observation consistent with the observation that hedgehog pathway activation parallels histologic activity in human NASH [9].

We and others have previously reported that hepatocyte cell death by apoptosis is a prominent feature of NASH [3], [4], [28], and consistent with the human disease, serum ALT values and TUNEL-positive liver cells were increased in mice fed the FFC diet. Vismodegib therapy reduced serum ALT values and TUNEL-positive liver cells. These hepatoprotective effects may be considered unexpected, as hepatocytes are thought to be unresponsive to hedgehog signaling [e.g., do not express Gli transcription factors in response to stress [29]] and, hence, would not be expected to respond directly to pharmacologic treatment with a smoothened inhibitor. However, we identified smoothened expression at both the mRNA and protein levels in isolated mouse hepatocytes, and demonstrated that vismodegib reduces DR5 mRNA expression in a cultured hepatoma cell line. Thus, hedgehog signaling pathways are likely operational in hepatocytes. Although hepatocytes do not express cilia which are thought to be essential for canonical hedgehog pathway signaling via the Gli transcription factors in mammalian cells [30], non-canonical hedgehog signaling has been observed in cells without cilia and occurs via a G-protein-dependent pathway independent of the Gli transcription factors [7]. Whether this non-canonical signaling pathway occurs in lipid laden hepatocytes has not been explored, but may be a potentially injurious pathway in this disease context.

The liver is particularly sensitive to apoptosis by death receptors [31]. Therefore, to further examine how vismodegib therapy reduced liver injury we profiled hepatic expression of the death receptors Fas, TNFR1 and DR5 (also termed TRAIL receptor-2) and the corresponding ligands. Although modest increases in Fas and TNFR1 and their cognate ligands were observed, the most striking increase was in DR5 expression. This observation is consistent with prior *in vitro* studies demonstrating an increase in DR5 expression in free fatty acid-treated hepatocytes, and the dependence upon DR5 for hepatocyte lipotoxicity by palmitate [16], [21]. To further extend these studies to an *in vivo* context, mice fed the FFC diet for three months were treated acutely with the agonistic anti-DR5 antibody MD5-1 (2 doses over 7 days). Interestingly, whereas the chow-fed animals displayed minimal hepatotoxicity following the MD5-1 administration, it markedly exacerbated liver injury in the FFC diet-fed mice as manifested by serum ALT values and TUNEL-positive liver nuclei. Pre-treatment with vismodegib for one week which reduces DR5 expression in FFC diet-fed mice also attenuated liver injury by MD5-1. These data raise the possibility that vismodegib reduces hepatocyte injury *in vivo* by directly or indirectly downregulating DR5 expression.

TRAIL is largely expressed by cells of the innate immune system including macrophages [6], and macrophages have been strongly implicated in lipotoxic disorders including NASH [22], [32]. Therefore, we examined markers for macrophages and their activation status in FFC diet-fed mice in the context of vismodegib therapy. Consistent with other studies, mice fed the FFC diet displayed increased macrophage markers in the liver including CD14, CD68, and F4/80 and more abundant Mac-2-positive cells. The macrophages appeared to be activated as TNF-α, IL-1β, IL-6 and MCP-1 were also increased in the mice on the FFC diet. This increase in activated macrophages was reduced by vismodegib therapy. Vismodegib may have reduced hepatic macrophage numbers and accumulation secondarily by directly reducing liver injury by TRAIL:DR5-mediated apoptosis and likely other mechanisms. Conversely, hepatocytes produce sonic hedgehog when injured such as has been documented in ballooned hepatocytes [8], and sonic hedgehog has been reported to be a chemoattractant factor in gastric inflammation [24]. Thus, vismodegib may also secondarily reduce liver inflammation in this murine model of NASH by preventing monocyte/macrophage infiltration into the liver.

In contrast to decreased liver injury, vismodegib did not prevent hepatic steatosis. The FFC diet induced a several fold increase in hepatic neutral triglycerides, which in fact was slightly accentuated in the vismodegib-treated animals. We do not consider this effect of vismodegib as a drawback since triglyceride synthesis has been suggested as a protective mechanism against lipotoxicity [33]. Moreover, as hepatic steatosis has been shown beneficial for liver regeneration after partial hepatectomy [34], we speculate that perhaps vismodegib may be a useful drug for patient with NASH undergoing hepatectomy.

Vismodegib treatment in FFC diet-fed mice also attenuates liver fibrosis. Multiple mechanisms may contribute to this observation. First, hedgehog signaling has been implicated in MCP-1 expression in the bile duct-ligated rodents [35]; and MCP-1-driven recruitment of hepatic macrophages contributes to liver fibrosis [36]. Second, prior publications have demonstrated that smoothened inhibition directly suppresses stellate cell activation and myofibroblast accumulation in the liver [10], [26], [37]. Finally, a reduction in hepatocyte injury would also be anticipated to reduce the inflammatory cascades promoting inflammation and fibrogenesis. A complete dissection of these mechanisms is beyond the scope of the current study, and likely, the antifibrogenic effect of vismodegib observed in our current studies is pleiotropic.

**Figure 7:**
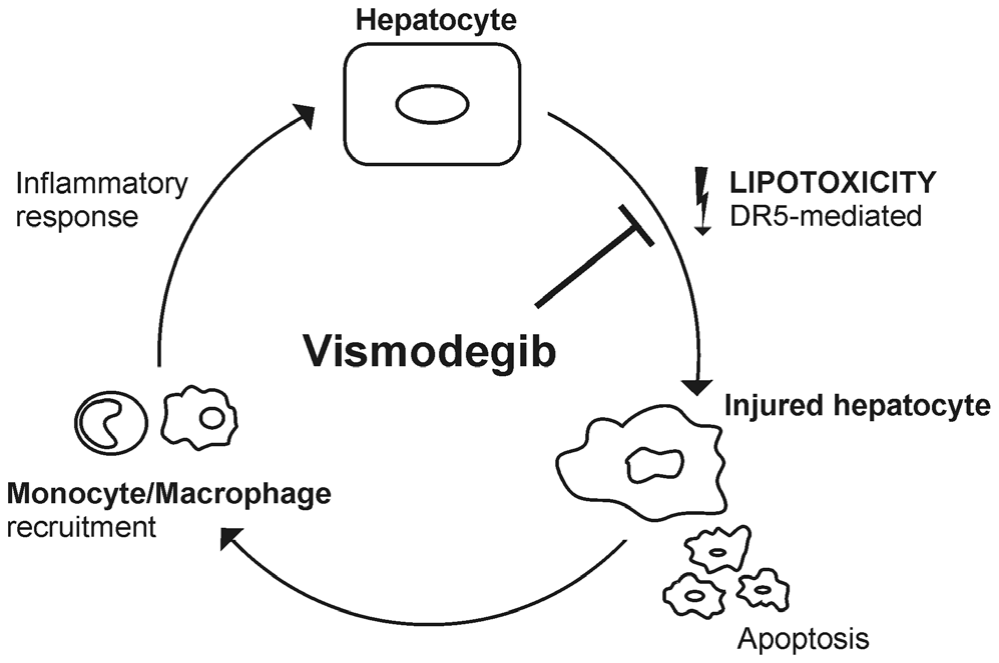
Schematic mechanistic representation for vismodegib therapy in NASH. Lipotoxicity during NASH induces TRAIL:DR5-mediated hepatocyte injury and apoptosis. Injured hepatocytes secrete chemoattractants, such as MCP-1 or sonic hedgehog, which attract and recruit monocytes. Monocytes/macrophages infiltrating the liver are activated and promote an inflammatory response, which further causes hepatocyte damage. Vismodegib disrupts this cycle by inhibiting upregulation of DR5 and thus abrogating TRAIL:DR5-mediated apoptosis and related liver injury, and consequent monocyte/macrophage recruitment to the liver.

In summary, our data extend prior observations regarding hedgehog pathway activation in liver injury by demonstrating a salutary effect of vismodegib in a preclinical model of NASH (Fig. 7). Our unique observations include an effect of vismodegib on modulating death receptor expression and signaling, especially DR5, and in suppressing inflammation by hepatic monocytes/macrophages. We propose that there is a feed-forward injurious loop in NASH whereby DR5-mediated lipotoxicity results in hepatocyte apoptosis and injury, secondarily attracting monocytes/macrophages to the liver which further cause hepatocyte damage. Vismodegib appears to interrupt this feed-forward loop by reducing both processes. Given the beneficial effects of short-term vismodegib administration in our murine model of NASH, we speculate that this pharmacologic agent or other inhibitors of hedgehog pathway activation could be salutary following chronic administration in human NASH.

## References

[1] Milic S, Stimac D (2012) Nonalcoholic fatty liver disease/steatohepatitis: epidemiology, pathogenesis, clinical presentation and treatment. Dig Dis 30: 158–162. 22722431 10.1159/000336669

[2] Alkhouri N, Carter-Kent C, Feldstein AE (2011) Apoptosis in nonalcoholic fatty liver disease: diagnostic and therapeutic implications. Expert Rev Gastroenterol Hepatol 5: 201–212. 21476915 10.1586/egh.11.6 PMC3119461

[3] Ribeiro PS, Cortez-Pinto H, Sola S, Castro RE, Ramalho RM, et al (2004) Hepatocyte apoptosis, expression of death receptors, and activation of NF-kappaB in the liver of nonalcoholic and alcoholic steatohepatitis patients. Am J Gastroenterol 99: 1708–1717. 15330907 10.1111/j.1572-0241.2004.40009.x

[4] Feldstein AE, Canbay A, Angulo P, Taniai M, Burgart LJ, et al (2003) Hepatocyte apoptosis and fas expression are prominent features of human nonalcoholic steatohepatitis. Gastroenterology 125: 437–443. 12891546 10.1016/s0016-5085(03)00907-7

[5] Strasser A, Jost PJ, Nagata S (2009) The many roles of FAS receptor signaling in the immune system. Immunity 30: 180–192. 19239902 10.1016/j.immuni.2009.01.001 PMC2956119

[6] Falschlehner C, Schaefer U, Walczak H (2009) Following TRAIL's path in the immune system. Immunology 127: 145–154. 19476510 10.1111/j.1365-2567.2009.03058.x PMC2691779

[7] Lin C, Nozawa YI, Chuang PT (2012) The path to chemotaxis and transcription is smoothened. Sci Signal 5: pe35. 22912492 10.1126/scisignal.2003423

[8] Rangwala F, Guy CD, Lu J, Suzuki A, Burchette JL, et al (2011) Increased production of sonic hedgehog by ballooned hepatocytes. J Pathol 224: 401–410. 21547909 10.1002/path.2888 PMC3628812

[9] Guy CD, Suzuki A, Zdanowicz M, Abdelmalek MF, Burchette J, et al (2012) Hedgehog pathway activation parallels histologic severity of injury and fibrosis in human nonalcoholic fatty liver disease. Hepatology 55: 1711–1721. 22213086 10.1002/hep.25559 PMC3499103

[10] Syn WK, Choi SS, Liaskou E, Karaca GF, Agboola KM, et al (2011) Osteopontin is induced by hedgehog pathway activation and promotes fibrosis progression in nonalcoholic steatohepatitis. Hepatology 53: 106–115. 20967826 10.1002/hep.23998 PMC3025083

[11] Swiderska-Syn M, Suzuki A, Guy CD, Schwimmer JB, Abdelmalek MF, et al (2013) Hedgehog pathway and pediatric nonalcoholic fatty liver disease. Hepatology 57: 1814–1825. 23300059 10.1002/hep.26230 PMC3637920

[12] Syn WK, Agboola KM, Swiderska M, Michelotti GA, Liaskou E, et al (2012) NKT-associated hedgehog and osteopontin drive fibrogenesis in non-alcoholic fatty liver disease. Gut 61: 1323–1329. 22427237 10.1136/gutjnl-2011-301857 PMC3578424

[13] Charlton M, Krishnan A, Viker K, Sanderson S, Cazanave S, et al (2011) Fast food diet mouse: novel small animal model of NASH with ballooning, progressive fibrosis, and high physiological fidelity to the human condition. Am J Physiol Gastrointest Liver Physiol 301: G825–834. 21836057 10.1152/ajpgi.00145.2011 PMC3220319

[14] Takeda K, Yamaguchi N, Akiba H, Kojima Y, Hayakawa Y, et al (2004) Induction of tumor-specific T cell immunity by anti-DR5 antibody therapy. J Exp Med 199: 437–448. 14769851 10.1084/jem.20031457 PMC2211825

[15] Canbay A, Feldstein AE, Higuchi H, Werneburg N, Grambihler A, et al (2003) Kupffer cell engulfment of apoptotic bodies stimulates death ligand and cytokine expression. Hepatology 38: 1188–1198. 14578857 10.1053/jhep.2003.50472

[16] Cazanave SC, Mott JL, Bronk SF, Werneburg NW, Fingas CD, et al (2011) Death receptor 5 signaling promotes hepatocyte lipoapoptosis. J Biol Chem 286: 39336–39348. 21941003 10.1074/jbc.M111.280420 PMC3234758

[17] Miao B, Zondlo S, Gibbs S, Cromley D, Hosagrahara VP, et al (2004) Raising HDL cholesterol without inducing hepatic steatosis and hypertriglyceridemia by a selective LXR modulator. J Lipid Res 45: 1410–1417. 15145986 10.1194/jlr.M300450-JLR200

[18] Canbay A, Higuchi H, Bronk SF, Taniai M, Sebo TJ, et al (2002) Fas enhances fibrogenesis in the bile duct ligated mouse: a link between apoptosis and fibrosis. Gastroenterology 123: 1323–1330. 12360492 10.1053/gast.2002.35953

[19] Kurita S, Mott JL, Almada LL, Bronk SF, Werneburg NW, et al (2010) GLI3-dependent repression of DR4 mediates hedgehog antagonism of TRAIL-induced apoptosis. Oncogene 29: 4848–4858. 20562908 10.1038/onc.2010.235 PMC2928864

[20] Cigna N, Farrokhi Moshai E, Brayer S, Marchal-Somme J, Wemeau-Stervinou L, et al (2012) The hedgehog system machinery controls transforming growth factor-beta-dependent myofibroblastic differentiation in humans: involvement in idiopathic pulmonary fibrosis. The American journal of pathology 181: 2126–2137. 23031257 10.1016/j.ajpath.2012.08.019

[21] Malhi H, Barreyro FJ, Isomoto H, Bronk SF, Gores GJ (2007) Free fatty acids sensitise hepatocytes to TRAIL mediated cytotoxicity. Gut 56: 1124–1131. 17470478 10.1136/gut.2006.118059 PMC1955518

[22] Osborn O, Olefsky JM (2012) The cellular and signaling networks linking the immune system and metabolism in disease. Nat Med 18: 363–374. 22395709 10.1038/nm.2627

[23] Baffy G (2009) Kupffer cells in non-alcoholic fatty liver disease: the emerging view. J Hepatol 51: 212–223. 19447517 10.1016/j.jhep.2009.03.008 PMC2694233

[24] Schumacher MA, Donnelly JM, Engevik AC, Xiao C, Yang L, et al.. (2012) Gastric Sonic Hedgehog acts as a macrophage chemoattractant during the immune response to Helicobacter pylori. Gastroenterology 142: 1150–1159 e1156. 10.1053/j.gastro.2012.01.029 PMC3335966 22285806

[25] Cullen SP, Henry CM, Kearney CJ, Logue SE, Feoktistova M, et al (2013) Fas/CD95-Induced Chemokines Can Serve as "Find-Me" Signals for Apoptotic Cells. Molecular Cell 49: 1034–1048. 23434371 10.1016/j.molcel.2013.01.025

[26] Philips GM, Chan IS, Swiderska M, Schroder VT, Guy C, et al (2011) Hedgehog signaling antagonist promotes regression of both liver fibrosis and hepatocellular carcinoma in a murine model of primary liver cancer. PLoS One 6: e23943. 21912653 10.1371/journal.pone.0023943 PMC3166282

[27] Pratap A, Singh S, Mundra V, Yang N, Panakanti R, et al (2012) Attenuation of early liver fibrosis by pharmacological inhibition of smoothened receptor signaling. J Drug Target 20: 770–782. 22994359 10.3109/1061186X.2012.719900

[28] Garcia-Monzon C, Lo Iacono O, Mayoral R, Gonzalez-Rodriguez A, Miquilena-Colina ME, et al (2011) Hepatic insulin resistance is associated with increased apoptosis and fibrogenesis in nonalcoholic steatohepatitis and chronic hepatitis C. J Hepatol. 54: 142–152. 10.1016/j.jhep.2010.06.021 20888662

[29] Bohinc BN, Diehl AM (2012) Mechanisms of disease progression in NASH: new paradigms. Clin Liver Dis 16: 549–565. 22824480 10.1016/j.cld.2012.05.002

[30] Roy S (2012) Cilia and Hedgehog: when and how was their marriage solemnized? Differentiation 83: S43–48. 22154138 10.1016/j.diff.2011.11.010

[31] Malhi H, Guicciardi ME, Gores GJ (2010) Hepatocyte death: a clear and present danger. Physiol Rev 90: 1165–1194. 20664081 10.1152/physrev.00061.2009 PMC2943859

[32] Olefsky JM, Glass CK (2010) Macrophages, inflammation, and insulin resistance. Annu Rev Physiol 72: 219–246. 20148674 10.1146/annurev-physiol-021909-135846

[33] Yamaguchi K, Yang L, McCall S, Huang J, Yu XX, et al (2007) Inhibiting triglyceride synthesis improves hepatic steatosis but exacerbates liver damage and fibrosis in obese mice with nonalcoholic steatohepatitis. Hepatology 45: 1366–1374. 17476695 10.1002/hep.21655

[34] Sydor S, Gu Y, Schlattjan M, Bechmann LP, Rauen U, et al (2013) Steatosis does not impair liver regeneration after partial hepatectomy. Laboratory investigation; a journal of technical methods and pathology 93: 20–30. 23069937 10.1038/labinvest.2012.142

[35] Omenetti A, Syn WK, Jung Y, Francis H, Porrello A, et al (2009) Repair-related activation of hedgehog signaling promotes cholangiocyte chemokine production. Hepatology 50: 518–527. 19575365 10.1002/hep.23019 PMC2722691

[36] Wynn TA, Barron L (2010) Macrophages: master regulators of inflammation and fibrosis. Seminars in liver disease 30: 245–257. 20665377 10.1055/s-0030-1255354 PMC2924662

[37] Michelotti GA, Xie G, Swiderska M, Choi SS, Karaca G, et al.. (2013) Smoothened is a master regulator of adult liver repair. The Journal of clinical investigation. In pres. 10.1172/JCI66904 PMC3668809 23563311

